# MnO_2_ Heterostructure on Carbon Nanotubes as Cathode Material for Aqueous Zinc-Ion Batteries

**DOI:** 10.3390/ijms21134689

**Published:** 2020-06-30

**Authors:** Sonti Khamsanga, Mai Thanh Nguyen, Tetsu Yonezawa, Patchanita Thamyongkit, Rojana Pornprasertsuk, Prasit Pattananuwat, Adisorn Tuantranont, Siwaruk Siwamogsatham, Soorathep Kheawhom

**Affiliations:** 1 Department of Chemical Engineering, Faculty of Engineering, Chulalongkorn University, Bangkok 10330, Thailand; 6071443721@student.chula.ac.th; 2 Division of Materials Science and Engineering, Faculty of Engineering, Hokkaido University, Hokkaido 060-8628, Japan; mai_nt@eng.hokudai.ac.jp (M.T.N.); tetsu@eng.hokudai.ac.jp (T.Y.); 3 Institute of Business-Regional Collaborations, Hokkaido University, Hokkaido 001-0021, Japan; 4 Department of Chemistry, Faculty of Science, Chulalongkorn University, Bangkok 10330, Thailand; patchanita.v@chula.ac.th; 5 Department of Materials Science, Faculty of Science, Chulalongkorn University, Bangkok 10330, Thailand; rojana.p@chula.ac.th (R.P.); prasit.pat@chula.ac.th (P.P.); 6 Center of Excellence in Petrochemical and Materials Technology, Chulalongkorn University, Bangkok 10330, Thailand; 7 Research Unit of Advanced Materials for Energy Storage, Chulalongkorn University, Bangkok 10330, Thailand; 8 National Science and Technology Development Agency, Pathumthani 12120, Thailand; adisorn.tua@nstda.or.th (A.T.); siwaruk.siw@nstda.or.th (S.S.)

**Keywords:** zinc-ion battery, cathode, manganese oxide, carbon nanotubes, heterostructure

## Abstract

Due to their cost effectiveness, high safety, and eco-friendliness, zinc-ion batteries (ZIBs) are receiving much attention nowadays. In the production of rechargeable ZIBs, the cathode plays an important role. Manganese oxide (MnO_2_) is considered the most promising and widely investigated intercalation cathode material. Nonetheless, MnO_2_ cathodes are subjected to challenging issues viz. limited capacity, low rate capability and poor cycling stability. It is seen that the MnO_2_ heterostructure can enable long-term cycling stability in different types of energy devices. Herein, a versatile chemical method for the preparation of MnO_2_ heterostructure on multi-walled carbon nanotubes (MNH-CNT) is reported. Besides, the synthesized MNH-CNT is composed of δ-MnO_2_ and γ-MnO_2_. A ZIB using the MNH-CNT cathode delivers a high initial discharge capacity of 236 mAh g^−1^ at 400 mA g^−1^, 108 mAh g^−1^ at 1600 mA g^−1^ and excellent cycling stability. A pseudocapacitive behavior investigation demonstrates fast zinc ion diffusion via a diffusion-controlled process with low capacitive contribution. Overall, the MNH-CNT cathode is seen to exhibit superior electrochemical performance. This work presents new opportunities for improving the discharge capacity and cycling stability of aqueous ZIBs.

## 1. Introduction

Metal-ion batteries (MIBs) are rechargeable batteries that use metal ions as a charge carrier being capable of reversible intercalation and deintercalation into the host material [1,2,3,4,5]. In recent years, on account of their low self-discharge, less memory effect and high efficiency, the implementation of MIBs for energy storage has become very popular [6,7]. Of different types of MIBs, lithium-ion batteries (LIBs) are widely used for various applications due to their high energy density and long cycle life [8,9]. However, some important factors such as high cost and safety issues tarnish the large-scale application of LIBs [10,11]. Besides LIBs, various monovalent (Na^+^, K^+^) and multivalent (Mg^2+^, Zn^2+^, Al^3+^) ions have been considered as charge carriers in MIBs. In view of this, ZIBs have attracted the interest of researchers [12,13,14,15]. It is noted that zinc (Zn) has a low redox potential of −0.763 V vs. a standard hydrogen electrode (SHE), which is favorable for near neutral or slightly acidic aqueous electrolytes [16]. In addition, Zn has a higher specific volumetric capacity compared to that of lithium viz. 5855 and 2066 mAh cm^−3^, respectively [17]. As compared to lithium, Zn is lower in cost and highly abundant [18,19].

A typical ZIB consists of a cathode (for hosting Zn ions), a Zn metal anode, an electrolyte, and a separator (to separate the cathode and anode) [20,21]. In general, the development of aqueous ZIBs has been limited by the cathode materials used [22,23,24]. In this respect, MnO_2_ has been found to be one of the most promising alternatives due to its low cost and good environmental compatibility along with high operating voltage and a theoretical capacity of 308 mAh g^−1^ [15,25,26,27]. Various types of MnO_2_ have been employed as cathode material for ZIBs such as α-MnO_2_ nanorods with onion-like carbon (OLC) (reported capacity of 168 mAh g^−1^ at 246 mA g^−1^) [8], δ-MnO_2_ nanosheets (reported capacity of 133 mAh g^−1^ at 100 mA g^−1^) [28], and MnO_2_ nanorods with graphene (reported capacity of 301 mAh g^−1^ at 500 mA g^−1^) [29]. In addition, MnO_2_ can exist in a variety of crystallographic polymorphs such as. α-, β-, γ-, δ- and λ-MnO_2_, depending on how MnO_6_ octahedral units are connected by different types of network. While α-, β- and γ-MnO_2_ have tunnel structures, δ-MnO_2_ has a layered structure [30]. Nevertheless, during cycling, MnO_2_ suffers poor capacity and inadequate performance because of its poor electronic conductivity and the instability of its electrode [22,31].

In order to solve these issues, graphite and carbon nanotubes (CNTs) have been introduced to support the MnO_2_ nanostructure because of their excellent electrical conductivity and high surface area [32,33,34,35]. Among conductive materials, CNTs including single- (SWCNTs) and multi-walled carbon nanotubes (MWCNTs) have been used as supporting materials to form composites with MnO_2_ [31]. Researchers have reported several significant results concerning MnO_2_ with CNTs. Chou et al. [36], for example, successfully electrodeposited MnO_2_ nanowires onto CNT paper. The composite CNT paper demonstrated good cyclability after 3000 cycles. For this reason, it acted as a conductive and active substrate for flexible electrodes of a supercapacitor. Wang et al. [34] synthesized a nanocomposite of MnO_2_/CNTs by direct redox reaction. The result indicated that capacitance retained more than 90% of initial capacitance after 2000 cycles because electrical conductivity influenced the specific capacitance.

Besides conductive carbon support, using a heterostructure is an attractive concept in that two types of materials and/or nanostructures have been directly grown on supporting material, such as binder-free electrodes [37,38,39]. The birnessite-type δ-MnO_2_ exhibited a favorable electrochemical performance due to its relatively large interlayer distance (70 Å) for energy storage applications [22,40]. Moreover, γ-MnO_2_, as the anode material of rechargeable LIBs, provided high initial reversible capacity [41]. It is challenging that the MnO_2_ heterostructure could lead to remarkable electrochemical properties as well as enhanced battery performance. Thus, the MnO_2_ heterostructure was synthesized leading to an improvement not only of the electrical conductivity of the cathode but also the capacity and cycling stability of ZIBs.

Herein, the in situ reduction of potassium permanganate (KMnO_4_) using MWCNTs as a supporting material has been carried out to produce MnO_2_ heterostructure on multi-walled carbon nanotubes (MNH-CNT). The heterostructure was controlled via the ratio of an initial amount of KMnO_4_ and MWCNTs. The as-prepared composite was examined to determine its physical characterization. Subsequently, the electrochemical properties and performances of ZIBs which used the MnO_2_ on multi-walled carbon nanotubes (MN-CNT) as the host material cathode, are investigated and discussed.

## 2. Results and Discussion

### 2.1. Material Characterization

#### 2.1.1. X-ray Diffraction (XRD)

In this study, MN-CNT was synthesized via thermal reaction. Accordingly, the growth process of MnO_2_ on MWCNTs is stated as in Equation (1) [27]:

(1) $$4{\mathrm{KMnO}}_{4}+4C+2H_{2}{\mathrm{SO}}_{4}\;\to 4{\mathrm{MnO}}_{2}+3{\mathrm{CO}}_{2}+2K_{2}{\mathrm{SO}}_{4}+2H_{2}O$$

The outer wall of MWCNTs was oxidized by KMnO_4_ under strong acid condition. Further, MnO_2_ materialized due to redox reaction in which KMnO_4_ acted as an oxidant. This process led to the homogenous coverage of MnO_2_ formed on the MWCNT’s surface.

**Figure 1:**
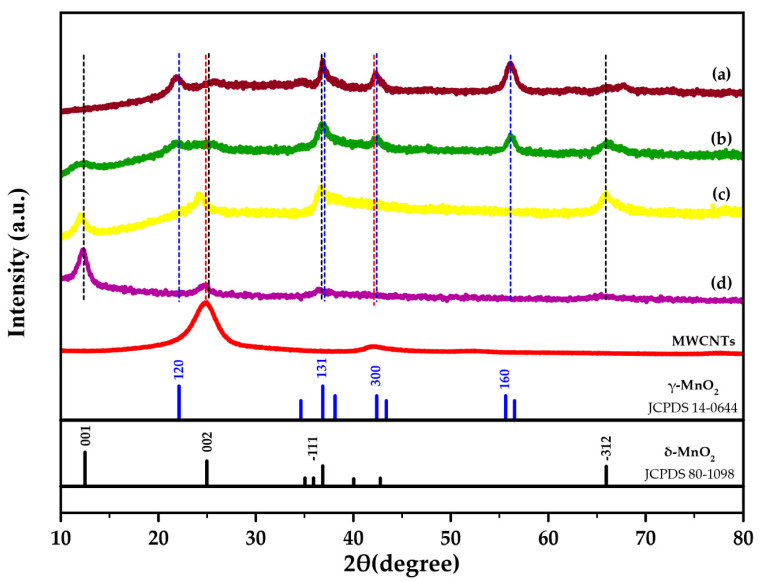
X-ray Diffraction (XRD) patterns of multi-walled carbon nanotubes (MWCNTs) and synthesized MnO_2_ on multi-walled carbon nanotubes (MN-CNT) with different ratios of MnO_2_:MWCNTs: (**a**) 60:40-MN-CNT6040 (**b**) 75:25-MN-CNT7525 (**c**) 90:10-MN-CNT9010 and (**d**) synthesized δ-MnO_2_.

XRD was conducted to study the crystal structure of the samples. In Figure 1, XRD patterns of pristine δ-MnO_2_, MWCNTs and MN-CNTs having different ratios of MnO_2_ and MWCNTs are shown. MN-CNT6040, MN-CNT7525 and MN-CNT9010 represent the MN-CNTs having MnO_2_ and MWCNT ratios of 60:40, 75:25 and 90:10, respectively. It can be seen that the pure MWCNTs show a sharp peak at around 26° and broad weak peaks at around 42°, which are characteristic of CNTs [35]. The four broad peaks of the MN-CNT9010 at around 12°, 24°, 37°, and 66° correspond to the crystal planes of (001), (002), (−111), and (−312) in δ-type MnO_2_ (JCPDS 80-1098), respectively [30,42]. They are similar to all the peaks of δ-MnO_2_. The broad peaks of the MN-CNT6040 at around 22°, 36.5°, 42°, and 56° correspond to the crystal planes of (120), (131), (300), and (160) in γ-type MnO_2_ (JCPDS 14-0644), respectively [41,42]. The MN-CNT7525 presents all the peaks of δ-type and γ-type MnO_2_, indicating that the heterostructure of MnO_2_ is growth with 75:25 ratio of MnO_2_ and MWCNT.

The weak-intensity diffraction peaks of MWCNTs on the XRD pattern of the MN-CNT may result from both the broad and high diffraction peaks of MnO_2_ at around 24° and 22° for δ- and γ-type MnO_2_, which defeats the signal of MWCNTs [36]. The calculated crystallite sizes of MN-CNT9010 and MN-CNT6040, obtained via Scherrer equation, are 7 and 12 nm, respectively. The calculated crystallite sizes of γ- and δ-MnO_2_ in MN-CNT7525 are 13 and 8 nm, respectively. The crystallite sizes of δ- and γ-MnO_2_ are the same when they are in the MnO_2_ heterostructure of MN-CNT. δ- and γ-MnO_2_ possess a layered and tunneled structure, respectively. It is suggested that foreign cations can insert/extract both a layered and tunneled structure, with the result that electrochemical performance can be enhanced by the MNH-CNT and thereby can be useful for energy storage applications [30].

#### 2.1.2. Field Emission Scanning Electron Microscope (FESEM)

**Figure 2:**
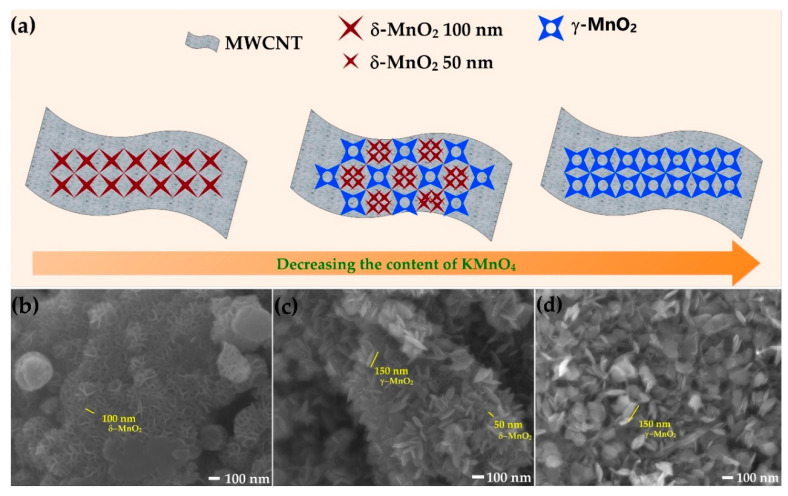
Morphological schema: (**a**) Schema of changes in morphology of MN-CNT followed by decreasing the content of KMnO_4_; (**b**) FESEM image of MN-CNT9010; (**c**) FESEM image of MN-CNT7525 and (**d**) FESEM image of MN-CNT6040.

In Figure 2a, the structure of MN-CNT, having different ratios of MnO_2_ and MWCNTs, is depicted and the corresponding morphological changes are shown. To determine the morphology of MN-CNTs, FESEM was carried out and the images are displayed in Figure 2b–d. MnO_2_ nanostructures are dispersed on the MWCNTs continuously. Besides, the type of MnO_2_ changed from δ- to γ-MnO_2_, as the amount of KMnO_4_ in the solution decreased. To be specific, the flower-like δ-MnO_2_ nanostructures, having petals about 100 nm in size in the MN-CNT9010 sample, change to the heterostructure of δ-MnO_2_ with petals being the size of 50 nm as well as leaf-like γ-MnO_2_ nanostructures having leaves around 150 nm in the MN-CNT7525 sample. It is demonstrated that, by limiting the amount of KMnO_4_, the δ-MnO_2_ cannot be completely generated. On the contrary, γ-MnO_2_ can form while the concentration of KMnO_4_ is still low during redox reaction. By continuing to decrease the amount of KMnO_4_ to 60% by weight, γ-MnO_2_ nanostructures can only be observed in the MN-CNT6040 sample. In Figure S1 of the supplementary file, lower magnification FESEM images of MN-CNT9010, MN-CNT7525, and MN-CNT6040 are presented. The MN-CNT, having two nanostructures of δ- and γ-MnO_2_ assists in increasing the contact area of the smaller petal size of δ-MnO_2_ between the electrolyte and cathode material, ensuring fast ion transfer in the charge/discharge process [43]. Besides, the γ-MnO_2_ in MNH-CNT is comprised not only of randomly arranged 1×1 and 1×2 tunnels, which are beneficial for Zn^2+^ ions intercalation/deintercalation but also accommodates the structural transformation from tunneled-type γ-MnO_2_ to the layered-type of Zn_y_MnO_2_. Such a randomly arranged structure can improve the stability of capacity during the cycling process [40].

In Figure S2 of the supplementary file, transmission electron microscope (TEM) images of MN-CNT having different ratios of MnO_2_ and MWCNTs are shown. In the case of MN-CNT6040 and MN-CNT7525, it is obvious that MnO_2_ formed on the MWCNTs. However, in the case of MN-CNT9010, MWCNTs cannot be seen clearly in the TEM image as a high amount of MnO_2_ was loaded. Thus, all parts of the MWCNTs were covered by MnO_2_. The transmission electron microscope with energy dispersive spectroscopy (TEM-EDS) (Figure S3 of the supplementary file) confirmed that carbon atoms of MWCNTs existed on MN-CNT9010. This proved to be in good agreement with XRD results (Figure 1), where the presence of MWCNTs in MN-CNT9010 was observed.

### 2.2. Electrochemical Performances

#### 2.2.1. Battery System

**Figure 3:**
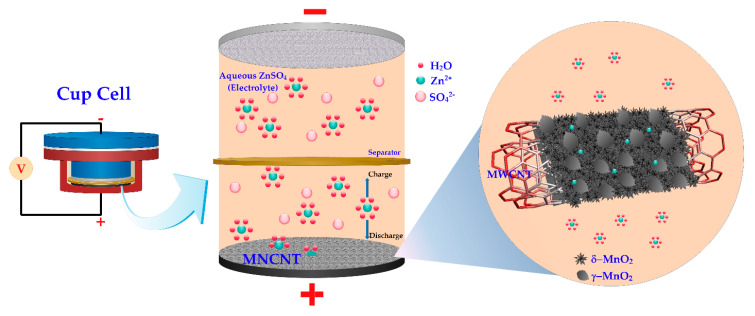
Schema of the chemistry of the zinc-ion battery. The inset on the right shows Zn^2+^ ion insertion into MnO_2_ heterostructure of MN-CNT.

In Figure 3, the battery configuration of the cup cell in this study, which is composed of the MNH-CNT electrode, zinc electrode, and ZnSO_4_ aqueous electrolyte, is shown. Anodic zinc, dissolved in the form of Zn^2+^ ions into an aqueous electrolyte containing Zn^2+^ ions, rapidly solvates in the form of solvated Zn^2+^ ions during the discharge process. Then, the solvated Zn^2+^ ions diffuse and pass through the separator to the MN-CNT electrode (cathode). The solvated Zn^2+^ ions are de-solvated in the form of Zn^2+^ ions [14,44] and intercalate into MnO_2_ heterostructure of MN-CNT, as illustrated by the inset in Figure 3. Further, an electron current starts to flow in the electrical loop from the electrical conduction of MWCNTs. These three processes can be reversed when the charging process occurs. Firstly, Zn^2+^ ions will de-intercalate from the MN-CNT electrode (anode).

Then, solvated species are formed. Lastly, Zn^2+^ ions are reduced to Zn and deposited back on the Zn electrode (cathode). The electrochemical reaction may be expressed as in Equation (2) for the Zn electrode and Equation (3) for the MN-CNT electrode:

(2) $$\mathrm{Zn}\;↔\mathrm{Zn}2^{+}+2e^{-}$$

(3) $$\mathrm{Zn}2^{+}+2e^{-}+(\delta +\gamma )-{\mathrm{MnO}}_{2}\;↔(\delta +\gamma )-{\mathrm{ZnMnO}}_{2}$$

During the electrochemical Zn^2+^ ion insertion, the layered-type δ-MnO_2_ structure can transform to spinel-type ZnMn_2_O_4_ with Mn(III) state and layered-type δ-Zn_x_MnO_2_ with Mn(II) state [15]. Meanwhile, the tunnel-type γ-MnO_2_ suffered a structural transformation to spinel-type Mn(III) phase (ZnMn_2_O_4_), tunnel-type γ-Zn*_x_*MnO_2_ and layered-type L-Zn*_y_*MnO_2_ [40].

#### 2.2.2. Electrochemical Performances

**Figure 4:**
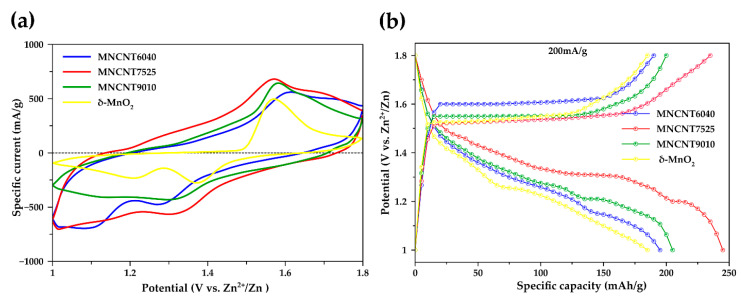
Electrochemical properties: (**a**) Cyclic voltammograms at a scan rate of 0.5 mV s^−1^; (**b**) Galvanostatic charge–discharge profile at 200 mA g^−1^ of the MN-CNT and δ-MnO_2_ cathode.

The electrochemical properties of δ-MnO_2_ and MN-CNT, having different ratios of MnO_2_ and MWCNTs cathodes, were evaluated by cyclic voltammetry (CV) within the potential range of 1.0–1.8 V vs. Zn/Zn^2+^ at a scan rate of 0.5 mV s^−1^. Figure 4a displays CV plots of their corresponding samples. Two distinct peaks of MN-CNT are observed: the cathodic peak at around 1.3 and 1.1 V and the anodic peak at 1.54 and 1.65 V. CV having two peaks, during discharge and charge, exhibits typical characteristics of the electrochemical insertion/extraction of Zn^2+^ ions in MnO_2_ structure [45,46,47]. According to previous studies, these peaks mainly suggest that the insertion/extraction of Zn^2+^ ions into/out of the interlayers of δ-MnO_2_ are associated with the reduction of Mn (IV) to Mn (III) and oxidation of Mn (III) to Mn (IV), respectively [22,40,48,49]. It is noted that the cathodic peak of MN-CNT7525 ∼ 1.1 V shifts to lower potential ∼ 1.05 V and the anodic peak of MN-CNT7525 ∼ 1.65 V shifts to higher potential ∼ 1.7 V. This may be affected by the insertion/extraction of zinc ions into/out of the γ-MnO_2_ of the MnO_2_ heterostructure of MN-CNT cathode.

In contrast, the cathodic peak of δ-MnO_2_ shifts to a higher potential and the anodic peak shifts to a lower potential. The CV curve of MN-CNT exhibits higher peak intensity and a larger enclosed area when compared with the δ-MnO_2_, indicating improved electrochemical performance and fast Zn^2+^ ion insertion/extraction in the cathode [50]. These results are consistent with the galvanostatic charge/discharge curves in Figure 4b which have two discharge plateaus at about 1.3 and 1.1 V. The discharge capacity for MN-CNT7525 is 245 mAh g^−1^ whereas the δ-MnO_2_, MN-CNT6040, and MN-CNT9010 register only 184, 193 and 202 mAh g^−1^, respectively. In addition, MN-CNT7525 shows a larger discharge plateau than that of δ-MnO_2_, MN-CNT6040, and MN-CNT9010, suggesting a higher capacity for Zn^2+^ ion insertion into the MnO_2_ heterostructure of MN-CNT than in the δ-MnO_2_ and single structure MnO_2_ of MN-CNT [15,51].

**Figure 5:**
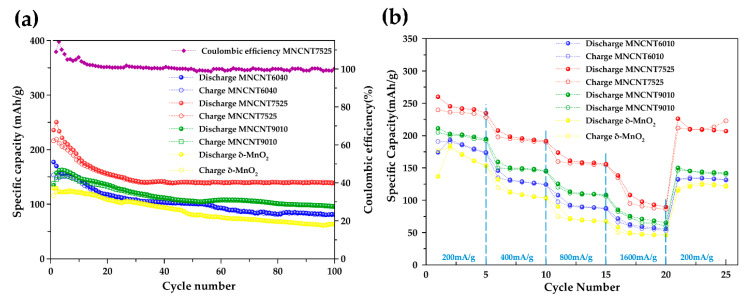
Performances of the batteries: (**a**) Cycling performance of the batteries at 400 mA g^−1^ and (**b**) Rate capability of the batteries at different discharge/charge rates.

In order to compare the battery performance of MN-CNT, having different ratios of MnO_2_ and MWCNTs, as the cathode for ZIBs, δ-MnO_2_ is used as a comparable cathode. Figure 5a displays the cycling behavior of the δ-MnO_2_ and MN-CNT electrodes along with the corresponding coulombic efficiency (CE) of MN-CNT7525, under the specific current density of 400 mA g^−1^ in the potential range of 1.0-1.8 V. The initial discharge capacities of MN-CNT6040, MN-CNT7525, MN-CNT9010 and δ-MnO_2_ are 177, 236, 135, and 129 mAh g^−1^, respectively.

The lower initial capacity of δ-MnO_2_ may result from its low intrinsic electronic conductivity because of the unstable state of Mn^3+^ during Zn-ion insertion [52]. This low capacity of the unsupported δ-MnO_2_ (pristine δ-MnO_2_) indicates that MWCNTs can enhance the electrical conductivity of the cathode’s active material. It is noted that the MN-CNT7525 exhibited the highest initial capacity. This high initial capacity may be attributed to the heterostructure of MnO_2_ on MWCNTs for Zn^2+^ insertion/extraction during the cycling process. Zn^2+^ ions can insert into both δ-MnO_2_, being smaller, nanoflower-like with a layered structure, and γ-MnO_2_ which is nanoleaf-like with a tunneled structure. Such a high initial capacity is superior to that of the δ-MnO_2_ cathode and most reported Zn-ion batteries, including onion-like carbon (OLC)-integrated α-MnO_2_ nanorods (168 mAh g^−1^ at 246 mA g^−1^) [8], δ-MnO_2_ nanosheets (133 mAh g^−1^ at 100 mA g^−1^) [28], and ZnMn_2_O_4_/Mn_2_O_3_ (82.6 mAh g^−1^ at 500 mA g^−1^) [53].

In the initial cycles, a gradual capacity fading was observed for the batteries having δ-MnO_2_, MN-CNT6040, and MN-CNT9010 electrodes. However, the battery using the MN-CNT7525 electrode showed a higher rate of capacity fading. This capacity fading may be attributed to the dissolution of MnO_2_ into the electrolyte during cycling. Moreover, in the case of MN-CNT7525, a higher dissolution was observed. After the initial 30 cycles, capacity fading was seen to slow down. This is probably because the equilibrium of Mn dissolution takes place from gradual Mn^2^^+^ ions’ dissolution in the electrolyte [54]. Interestingly, around the 20th cycle, the capacity of the MN-CNT7525 electrode starts to decrease gradually. Subsequently, after the 30th cycle, a stable capacity is observed. The capacity of the δ-MnO_2_, MN-CNT6040, and MN-CNT9010 electrodes, however, continuously decrease until the 100th cycle. The better performance could be due to the structural transformation between δ- and γ-MnO_2_ of MNH-CNT [15]. After 100 cycles, the MN-CNT7525 electrode delivers a capacity of 140 mAh g^−1^, CE being around 100%, indicating its good reversibility during the charging/discharging process. In contrast, the δ-MnO_2_, MN-CNT6040, and MN-CNT9010 cathodes deliver capacities of 63, 81, and 96 mAh g^−1^, respectively.

In Figure 5b, rate performances of the δ-MnO_2_ and MN-CNTs host material cathodes are displayed. Cycling takes place at various specific current densities of 200, 400, 800 and 1600 mA g^−1^ having five cycles for each rate. The rate performance of MN-CNT7525 was significantly higher than those of δ-MnO_2_, MN-CNT6040, and MN-CNT9010. The MN-CNT7525 cathode can be charged and discharged at a high rate of 1600 mA g^−1^, leading to a discharge and charge capacity of 108 and 95 mAh g^−1^, respectively. It is indicated that the MnO_2_ heterostructure of MN-CNT with its small nanoscale morphology of δ-MnO_2_ nanoflower-like and short-length crystal structure of γ-MnO_2_ provides a faster ion diffusion rate at a high current density. When the current turns back to 200 mA g^−1^, the MN-CNT7525 cathode can deliver both discharge and charge capacity of 226 and 212 mAh g^−1^, respectively. It is clear, therefore, that the MnO_2_ heterostructure of MN-CNT can improve not only cycling stability but also the rate performance for ZIBs. This behavior indicates that the MNH-CNT cathode can well be considered as Zn^2+^ ion storage material [52].

#### 2.2.3. Pseudocapacitive Behavior

**Figure 6:**
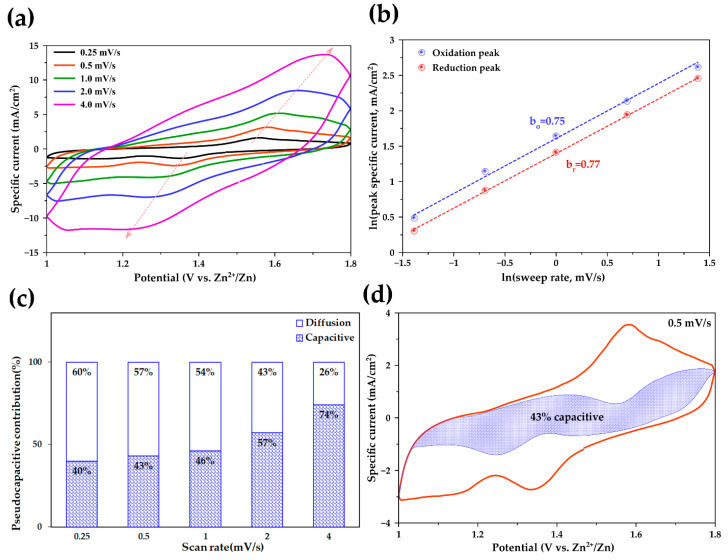
Electrochemical behavior: (**a**) Cyclic voltammograms of MN-CNT7525 cycling at different scan rates; (**b**) Analysis of *b*-value for oxidation and reduction peaks; (**c**) Capacitive contribution ratio of MN-CNT7525 electrode at different scan rates and (**d**) Capacitive contribution at a scan rate of 0.5 mV s^−1^.

To further investigate the electrochemical storage mechanism of the MN-CNT7525 electrode, CV curves at different scan rates between 0.25 and 4.0 mV s^−1^ in the voltage range 1.0–1.8 V were recorded and shown, as in Figure 6a. It is seen that one maximum reduction peak and oxidation peak distinctly exist in each cycle, with an increase in specific current exhibited, as scan rates are raised. At much higher scan rates, redox peaks are still maintained at an increased specific current. According to previous studies, the peak current (*i*) of the CV curves allows a power-law relationship with the scan rate (*v*) and can be used to analyze the charge storage mechanism, as in Equation (4) [55]:

(4) $$i=av^{b}$$

where *i* and *v* are the peak specific current and scan rate, respectively, and *a*, *b* are adjustable parameters. Equation (4) can be converted to logarithmic form, as in Equation (5):

(5) lni=blnv+lna

In Figure 6b, the plots of ln *i* vs ln *v* for both oxidation and reduction peaks are shown. The *b*-value denotes the slope of the plots. If the *b*-value is approximately equal to 1, the electrochemical system shows pseudocapacitive behavior controlled by a surface faradic reaction, whereas if the *b*-value is approximately equal to 0.5, typical ionic diffusion dominates the charge/discharge process by cation intercalation [56]. With scan rates ranging from 0.25 to 4 mV s^−1^, the peak specific current increases linearly with the increase in scan rate. The *b*-values for the peaks of *b*_o_ (oxidation process) and *b*_r_ (reduction process) are 0.75 and 0.77, respectively. It is indicated that the redox processes of MN-CNT7525 dominated both pseudocapacitive and diffusion kinetics. That is, the insertion/extraction of Zn^2+^ ions occurred not only on the surface but also took place at the pores inside. These results confirm the pseudocapacitive behavior of MN-CNT7525, leading to fast Zn^2+^ intercalation/extraction and excellent long-term cycling stability. Such an outcome may occur with the heterostructure of MnO_2_ on MWCNTs, demonstrating the material’s suitability as cathode material for ZIBs.

To quantitatively distinguish the capacitive contribution from the current response, the equation can be rewritten as in Equation (6) [57]:

(6) i=kv1+k2v12

where *k_1_* and *k_2_* are constants. Thus, the redox reaction is limited by the diffusion-controlled behavior; the current *i* at a fixed potential varies as *k*_2_*v*^1/2^. The capacitive contribution suggests that the peak current *i* varies as *k_1_v* [58,59,60]. By plotting *i*/*v*^1/2^ vs *v*^1/2^, *k_1_* and *k_2_* are calculated from the slope and the y-axis intercept at the point of a straight line, respectively.

In Figure 6c, the ratio of the pseudocapacitive contribution at various scan rates can be quantitatively determined, and the results are displayed. The capacitive contributions of MN-CNT7525 are 40%, 43%, 46%, 57%, and 74% at scan rates of 0.25, 0.5, 1.0, 2.0, and 4.0 mV s^−1^, respectively. It can be seen that, as scan rates increase, the capacitive contribution further increases. These results are consistent with the b-values obtained which are around 0.7 for MN-CNT7525.

In Figure 6d, the detail of the pseudocapacitive fraction at a scan rate of 0.5 mV s^−1^ is presented; 43% of the total charge, denoted by the blue shaded region, comes from capacitive processes. The low capacitive contribution is attributed to the fast ion diffusion of the MN-CNT7525 electrode, which leads to a high capacity and good cycling stability of the battery.

## 3. Materials and Methods

### 3.1. Materials

Sulfuric acid (H_2_SO_4_, 95%), zinc sulfate heptahydrate (ZnSO_4_⋅7H_2_O, 95%), carboxymethylcellulose sodium salt (CMC, 93%) and Pottasium permanganese (KMnO_4_, 99.3%) were supplied by Fujifilm Wako Pure Chemical Corporation (Osaka, Japan). MWCNTs (cocoon type with outer diameter 40-60 nm, 90%) were purchased from Suzhou Tanfeng Graphene Technology Co., Ltd. (Jiangsu, China). Manganese sulfate monohydrate (MnSO_4_⋅H_2_O, 99%) was purchased from Ajax Finechem Pty. Ltd. (Sydney, Australia). Nickel (Ni) foam (0.5 mm thick, 100 PPI) was purchased from Qijing Trading Co., Ltd. (Wenzhou, China). Double ring qualitative, Fast101 filter paper was purchased from GE Healthcare (Chicago, IL, USA). Carbon black (Vulcan® XC-72, 99.99%) was supplied by Cabot Corporation (Boston, MA, USA). Graphite foil was purchased from Shenzhen 3KS Electronic Material Co. Ltd. (Shenzhen, China). Zinc sheet (99.99%) was purchased from Sirikul Engineering Ltd., Part. (Samutprakan, Thailand).

### 3.2. Preparation of δ-MnO_2_ and MnO_2_ Heterostructure/MWCNTs (MNH-CNT)

The compared δ-MnO_2_ nanoparticles were synthesized by dissolving 1.98 g of KMnO_4_ in 60 mL of deionized (DI) water. Next, 0.336 g of MnSO_4_⋅H_2_O was dissolved in 20 mL of DI water. Then, the MnSO_4_⋅H_2_O solution was added dropwise to KMnO_4_ solution, and continuous stirring followed for 30 min. Afterwards, the mixture was transferred into a 100-mL Teflon autoclave and kept at 160 °C for 24 h in an oil bath. The product was dried at 80 °C for 12 h. It was duly collected and washed with DI water several times. MN-CNT was synthesized via thermal reaction. Then, 300 mg of MWCNTs were ground together in a mortar with KMnO_4_. The amount of KMnO_4_ was varied (1.35, 1.95, and 2.85 g) to provide different ratios of MnO_2_ and MWCNTs by 60:40 (MN-CNT6040), 75:25 (MN-CNT7525), and 90:10 (MN-CNT9010), respectively. Next, the mixed powder was dispersed in 300 mL of water stirring for 10 min. A total of 0.5 mL of concentrated H_2_SO_4_ was added to the above mixture with an additional 30 min of stirring. After that, the mixed solution was heated in an oil bath at 80 °C with continuous magnetic stirring for 1 h. The precipitate was washed and collected by centrifuging repeatedly with deionized water after the mixture was cooled to room temperature. Then, the solid product was dried in a vacuum at 50 °C for 24 h to obtain MN-CNT composite.

### 3.3. Electrodes and Cup Cell Preparation

The cathode using MN-CNT was prepared by mixing 70% wt. of MN-CNT, 20% wt. of carbon black, and 10% wt. of carboxymethylcellulose sodium salt binder. For comparison, the cathode of δ-MnO_2_ was fabricated the same way using pristine δ-MnO_2_ instead of MN-CNT. DI water was used to adjust the viscosity of the slurries. Each mixed slurry was stirred for 24 h at room temperature. Then, it was coated on a graphite foil current collector using a doctor blade and dried at 50 °C under vacuum before it was pressed by hydraulic press at 5 kPa for 1 min. The cathode contained about 5 mg of MN-CNT. Zinc anode was prepared by electrodeposition of zinc from ZnSO_4_ (0.5 M) aqueous solution onto Ni foam using zinc sheet as a counter electrode at current density of 60 mA cm^−2^. The amount of Zn deposited was 20 mg cm^−2^. Both the cathode and anode were punched into a 16-mm-diameter disk. The filter paper was punched into a 20-mm disk and used as the separator. Then, the cathode, anode and separator were fabricated in a cup cell by using 0.3 mL of aqueous ZnSO_4_ (1 M) as the electrolyte.

### 3.4. Materials Characterization

The phase states of MN-CNT were analyzed using X-Ray Diffraction (XRD, Rigaku Model Miniflex II, Tokyo, Japan) of the powder samples with Cu Kα radiation, λ = 1.5418 Å at a scanning range of 5-90°. Field emission-scanning electron microscope (FESEM, JEOL JSM-6701F, Tokyo, Japan) was used to observe the morphological image of MN-CNT. Transmission electron microscope with energy dispersive spectroscopy (TEM-EDS, JEOL JEM-2000FX, Tokyo, Japan) was used to prove the presence of MWCNTs in MN-CNTs.

### 3.5. Electrochemical Performances

Electrochemical measurements were carried out using a cup cell. CV was performed by the electrochemical measurement system (HZ-5000, Hokuto Denko, Tokyo, Japan) at a scan rate of 0.5 mV s^−1^ in the voltage range 1.0–1.8 V versus Zn^2+^/Zn. A battery testing system (EF-7100P, Electrofield, Osaka, Japan) was used to investigate the performance of the battery. The CVs for investigating capacitive behavior were tested at scanning rates from 0.25 to 4.0 mV s^−1^ between 1.0 and 1.8 V.

## 4. Conclusions

It is evident that the MnO_2_ heterostructure on MWCNTs proved to be a high-performance cathode for aqueous ZIBs. The incorporation of MWCNTs was found to improve the conductivity of MnO_2_. The formation of the δ-MnO_2_/γ-MnO_2_ heterostructure on MWCNTs not only enhanced specific capacity but also improved cycling stability of the MnO_2_ cathode. Consequently, the battery utilizing the MNH-CNT cathode demonstrated high discharge capacity, high-rate capability, as well as impressive cycling stability, exhibiting its superiority to the pristine δ-MnO_2_ electrode. Additionally, the capacitive contribution ratio of 43% at scan rate 0.5 mV s^−1^, reflecting the diffusion-controlled process, played a dominant role in the pseudocapacitive behavior of the MNH-CNT cathode. This work highlights the design of MnO_2_ heterostructure on CNTs for high-performance aqueous ZIBs.

## Supplementary Materials

Supplementary materials can be found at https://www.mdpi.com/1422-0067/21/13/4689/s1. Figure S1. FESEM images at low magnification of (a) MN-CNT9010; (b) MN-CNT7525 and(c) MN-CNT6040. Figure S2. TEM images of (a) MN-CNT9010 (b) MN-CNT7525 and(c) MN-CNT6040. Figure S3. TEM-EDS of (a) MN-CNT9010 (b) MN-CNT7525 and(c) MN-CNT6040.

## Abbreviations

ZIB	Zinc-ion battery
MNH-CNT	MnO_2_ heterostructure on multi-walled carbon nanotubes
MN-CNT	MnO_2_ on multi-walled carbon nanotubes
MN-CNT6040	MN-CNT having MnO_2_ and MWCNT ratio of 60:40
MN-CNT7525	MN-CNT having MnO_2_ and MWCNT ratio of 75:25
MN-CNT9010	MN-CNT having MnO_2_ and MWCNT ratio of 90:10
MIB	Metal-ion battery
LIB	Lithium-ion battery
SHE	Standard hydrogen potential
CNT	Carbon nanotube
SWCNT	Single-walled carbon nanotube
MWCNT	Multi-walled carbon nanotube
XRD	X-ray diffraction
FESEM	Field emission scanning electron microscope
TEM-EDS	Transmission electron microscope with energy dispersive spectroscopy
CV	Cyclic voltammetry
CE	Coulombic efficiency
OLC	Onion-like carbon
DI water	Deionized water
