# Supplementary material for: MnO2 Heterostructure on Carbon Nanotubes as Cathode Material for Aqueous Zinc-Ion Batteries
Source: Int J Mol Sci. 2020 Jun 30;21(13):4689. doi: 10.3390/ijms21134689 (PMC7369720; doi:10.3390/ijms21134689)
Supplement: Supplementary file 1 [file ijms-21-04689-s001.pdf]

Supplementary Information

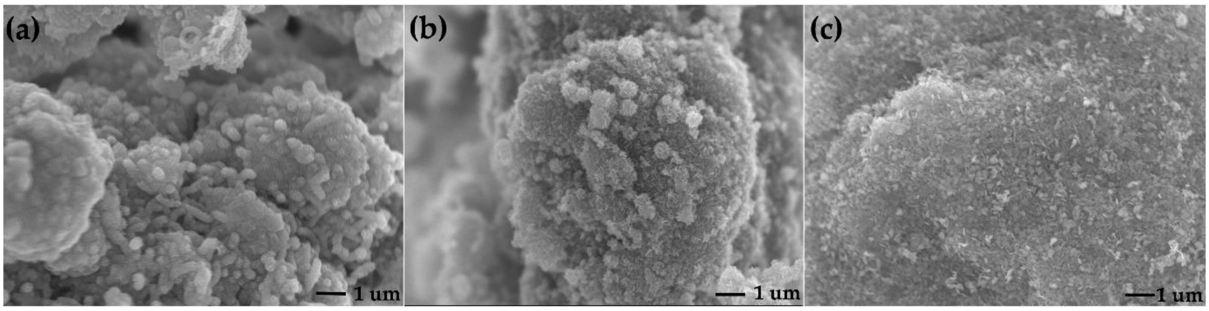

Figure S1. FESEM images at low magnification of (**a**) MN-CNT9010; (**b**) MN-CNT7525 and(**c**) MN-CNT6040

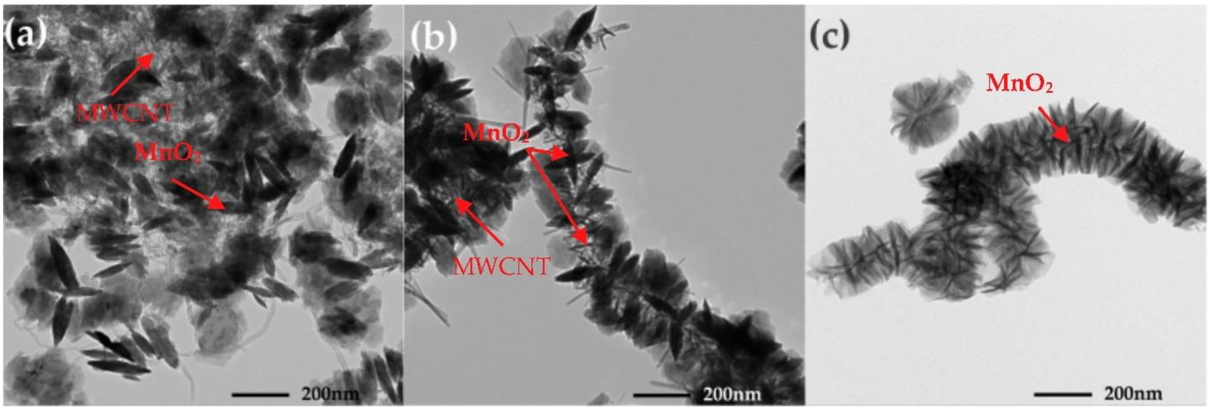

Figure S2. TEM images of (**a**) MN-CNT9010 (**b**) MN-CNT7525 and(**c**) MN-CNT6040

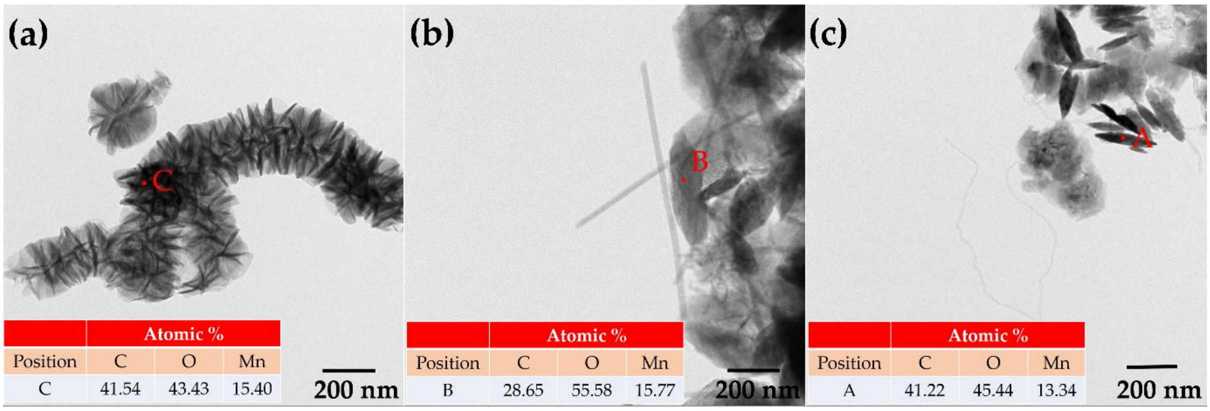

Figure S3. TEM-EDS of (a) MN-CNT9010 (b) MN-CNT7525 and(c) MN-CNT6040
